# Chitin Adsorbents for Toxic Metals: A Review

**DOI:** 10.3390/ijms18010114

**Published:** 2017-01-07

**Authors:** Ioannis Anastopoulos, Amit Bhatnagar, Dimitrios N. Bikiaris, George Z. Kyzas

**Affiliations:** 1 Laboratory of Soils and Agricultural Chemistry, Department of Natural Resources and Agricultural Engineering, Agricultural University of Athens, GR-118 55 Athens, Greece; 2 Department of Agrobiotechnology, Agricultural Research Institute, P.O. Box 22016, 1516 Nicosia, Cyprus; 3 Department of Environmental and Biological Sciences, University of Eastern Finland, P.O. Box 1627, FI-70211 Kuopio, Finland; amit.bhatnagar@uef.fi; 4 Division of Chemical Technology, Department of Chemistry, Aristotle University of Thessaloniki, GR-54124 Thessaloniki, Greece; dbic@chem.auth.gr; 5 Hephaestus Advanced Laboratory, Eastern Macedonia and Thrace Institute of Technology, GR-65404 Kavala, Greece

**Keywords:** chitins, adsorption, heavy metals, isotherms, thermodynamics

## Abstract

Wastewater treatment is still a critical issue all over the world. Among examined methods for the decontamination of wastewaters, adsorption is a promising, cheap, environmentally friendly and efficient procedure. There are various types of adsorbents that have been used to remove different pollutants such as agricultural waste, compost, nanomaterials, algae, etc., Chitin (poly-β-(1,4)-*N*-acetyl-d-glucosamine) is the second most abundant natural biopolymer and it has attracted scientific attention as an inexpensive adsorbent for toxic metals. This review article provides information about the use of chitin as an adsorbent. A list of chitin adsorbents with maximum adsorption capacity and the best isotherm and kinetic fitting models are provided. Moreover, thermodynamic studies, regeneration studies, the mechanism of adsorption and the experimental conditions are also discussed in depth.

## 1. Introduction

One of the biggest problems of recent times is the management/treatment of wastewaters, especially those originating from metal industries. These types of effluents have big concentrations of heavy metals (ionic forms). Examples of the metals that exist in these effluents are Cd, Zn, Pb, Cr, Ni, Cu, V, Pt, Ag, Ti, etc., and they mainly come from different industries. Many applications produce metal effluents, such as anodizing-cleaning, electroplating, conversion-coating, milling, electroless depositions, and etching [1].

One very hazardous metal is chromium. It is dangerous to living orgasms including humans, but chromium is considered to be less toxic than Cd, Pb and Hg. Another widely used metal in the industries (metal plating) is cadmium. It exists in waters/effluents and mainly in mining wastes. It has similar properties to Zn, and for this reason, they frequently undergo geochemical processes together. Their oxidation state in water is +2. Some disastrous results for humans exposed to these metals are high blood pressure, destruction of red blood cells, destruction of testicular tissue, and kidney damage. Also, Cd can replace Zn in some biochemical enzymes, altering their stereostructure and impairing their catalytic activity. These metals are very common in water and sediment pollutants in harbors surrounded by industrial facilities.

Special mention is necessary about the very toxic and hazardous ion cyanide (CN^−^). It is considered to be a poisonous substance and it exists in the form of HCN in waters. CN^−^ can form some less toxic ions with Fe(II) such as Fe(CN)_6_^4−^ due to its high affinity. Hydrocyanide is used in gas chamber executions, which mainly happens in the USA. Many industries worldwide, in particular metal cleaning or electroplating or even some mineral processing operations, also use this ion (CN^−^). Cyanide ion is a major part of the effluents originating from gas works and generally coke ovens.

Based on the above, it is necessary to carefully treat and remove the effluents containing heavy metals. One of the most trendy and promising techniques is considered to be adsorption due to its simplicity, low cost and reuse potential [2,3,4,5,6,7,8,9,10,11,12,13,14,15]. In this review article, a screening of the recent literature will be done regarding the use of chitin adsorbents as potential materials for the removal of toxic metals. In the past, many papers were published emphasizing other applications of chitin (in biomedicine, tissue engineering, scaffolds, etc.) [16,17,18,19,20].

**Figure 1:**
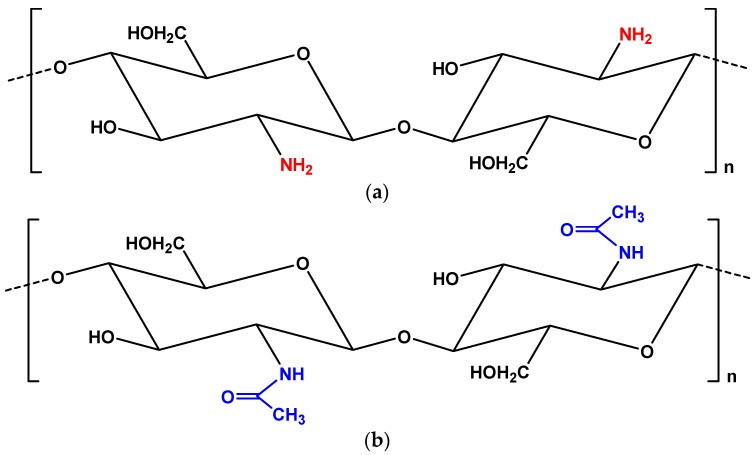
Chemical structure of (**a**) chitosan and (**b**) chitin.

It is a fact that in adsorption technology, two adsorbent materials cannot be compared (even for the same pollutant) without keeping the same experimental conditions. Some of the basic parameters which strongly influence the whole procedure are (i) the pH solution; (ii) the contact time; (iii) the initial pollutant’s concentration; (iv) the temperature; (v) the agitation speed; (vi) the volume of the adsorbate; (viii) the ionic strength of the solution and (ix) the adsorbent’s dosage, etc. It is clear that if any of the aforementioned conditions varies, the experiment will not be the same and consequently no comparison will be valid. Therefore, for that reason, the only (and mostly the “safest”) comparison that can be achieved is for adsorbent/adsorbate systems of the same study. On the other hand, it is true that the majority of review articles cannot clearly criticize the published articles due to the difficulty of the aforementioned reason. Another important point of the present review article is that until now, no other review/overview/summary was published regarding the removal of heavy metals with adsorption onto chitin derivatives. Numerous researchers have published review articles on topics such as the preparation and application of chitosan adsorbents (grafted, cross-linked and generally modified) for the removal of various pollutants [21,22,23,24,25,26,27,28]. Chitosan is considered to be more “flexible” from a structural point of view due to its amino groups (making it easy to modify) (Figure 1a). However, chitin (Figure 1b), which is the origin material of chitosan, is cheaper; the latter must be highlighted.

## 2. Chitin

**Figure 2:**
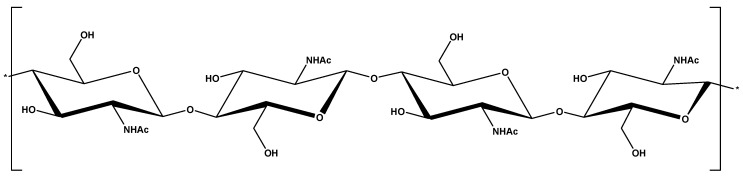
Chemical structure of chitin.

It is redundant to repeat theories about the structure of chitin, etc. However, it is mandatory to review some crucial characteristics of this material. So, chitin(C_8_H_13_O_5_N)_n_ is considered to be second most abundant biopolymer worldwide coming from a renewable organic resource. It is a polysaccharide with a long polymeric chain, and due to this it has many advantages for modification during the synthesis of derivatives. From a structural point of view, chitin is a polymer with a high molecular weight, linear sequential units of *N*-acetyl-d-glucosamine (linked by b-1,4 units) and it chemically resembles cellulose, in which the hydroxyl group at carbon-2 in cellulose has been substituted by an acetamido group. In general, chitin can be found in powder form or even in granules (or sheets). The initial form of chitin (pure) is not expensive and is easily available on the commercial market. The origin of the word “chitin” is from the Greek word “chiton’’, which means a coat. Braconnot (1811) used chitin for the first time. Chitin can be purchased at a low cost/price from organic sources (especially from krill, lobsters, crabs and shrimps) or wastes derived from some industries of seafood processing. Chitin is classified in natural polymers as having very good properties (biocompatibility/biodegradability, non-toxicity, etc.) (Figure 2).

## 3. Removal of Toxic Metals

Karthikeyan et al. [29] tested the removal of Fe^3+^ by chitin. A particle size of 0.21 mm exhibited higher removal efficiency than sizes of 0.50 and 0.71 mm due to the higher surface area. Kinetic studies showed that equilibrium was observed after 8 min. An inhibition of adsorption was noticed by the presence of chloride together with Fe^3+^. More specifically, as the chloride ion concentration increased from 0 to 1000 mg/L, the removal percentage of Fe^3+^ decreased from 64.5% to 27.65%. One possible explanation is that the greater the chloride ion concentration, the greater the formation of soluble chloro-complexes and the less free Fe^3+^ there is. These chloro-complexes have less affinity than free metal ions towards chitin. Similarly, in the presence of nitrate, the Fe^3+^ prefers to form complexes that are adsorbed onto the chitin surface. Regarding the effect of the ionic strength, the presence of perchlorate up to 750 mg has no effect on iron adsorption. Only at a high concentration of 1000 mg/L did a reduction of the iron adsorption percentage occur. The effect of co-ions such as Cu^2+^, Zn^2+^ and Cr^6+^ on iron uptake demonstrated that an equal amount of Cu^2+^ had a negative effect on the adsorption rate while Zn^2+^ and Cr^6+^ had a lesser effect. As the temperature rose from 20 to 50 °C, the iron removal was increased due to the enlargement of the pore size and/or the activation of the adsorbent surface and the increase in the mobility of the metal ions. Based on thermodynamic studies, adsorption proved to be spontaneous and endothermic with positive entropy change values.

The chitin of pink shrimp was explored in capturing Pb^2+^ from aqueous solutions [30]. The maximum adsorption was estimated to be 99.7% at pH 9, with 200 min of contact time, 5 g/L of biosorption dosage and an the initial lead concentration 20 mg/L, a temperature of 30 °C and a 200 rpm agitation speed.

The adsorption of Pb^2+^ and Cd^2+^ by chemically modified chitin with polypyrrole (PPy-g-Ch) was examined [31]. FTIR analysis showed the significant alteration of bands before and after adsorption. More specifically, after adsorption, the peak that appeared at 3436.53 cm^−1^ in PPy-g-Ch was shifted to 3476.41 cm^−1^ for Pb-loaded PPy-g-Ch and to 3455.36 cm^−1^ for Cd-loaded PPy-g-Ch, thus indicating the participation of –OH and –NH groups in the uptake of metals. The maximum adsorption was found to occur at pH 6 and equilibrium was achieved within 60 min. Thermodynamic parameters were estimated and revealed the endothermic, spontaneity and the physisorption characteristics of the adsorption process.

Xiong examined the use of chitin for Cd^2+^ adsorption [32]. The maximum removal was obtained at pH 5.41. The increase of the temperature from 288 to 318 K was found to increase the uptake efficiency from 87.1 to 102 mg/g. FTIR spectra before and after Cd^2+^ adsorption showed that the acetylamino and hydroxyl groups were involved in the metal removal. The Gibbs free energy was calculated to be negative, thus demonstrating the feasibility and spontaneity of the adsorption. The activation energy was 63.1 kJ/mol and it lies in the range of 40–800 kJ/mol, indicating chemisorption. The removal of Cd^2+^ by using the chitin/polyethylene glycol binary blend was also tested by Mohan and Syed Shafi [33]. The metal uptake was found to be affected by changing the pH from 4 to 8. More specifically, the optimum pH value was noticed at pH 5.5, while for pH values higher than 5.5, the adsorption reduced due to the precipitation of Cd^2+^ as Cd(OH)_2_. Equilibrium was achieved at 210 min of contact time. Calcareous chitin was fabricated by the alkaline treatment of crustacean exoskeleton to produce a porous matrix of chitin and calcium carbonate free of original proteins in order to explore Cd^2+^ uptake [34]. Compared to chitin, calcareous chitin (CaCh) gave a higher adsorption of about 2.3 times greater than chitin. One possible explanation is the synergistic effect on adsorption of both chitin and calcium carbonate, and secondly due to the higher specific area. Based on the abundance of raw materials and the minimum handling processing without risks, the cost of the CaCh is estimated to be between $5 and $7 per kg (NaOH 4% which may be recycled, 60 °C, 45 min).

Chitin (CH) powder was extracted in the laboratory from yellow lobster waste and was used for the sequestration of Cu^2+^ from aqueous media [35]. The highest adsorption was observed at pH 4 and intraparticle diffusion participated in the adsorption process, but it was not the only rate-limiting step. The Gibbs free energy was estimated to be negative at 25 °C, demonstrating the spontaneity of the adsorption process, whereas it was negative at 35 and 45 °C, suggesting that the Cu^2+^ uptake process was not feasible at these temperatures. Standard enthalpy was calculated to be negative which reveals the exothermic and physical characteristics of adsorption. Desorption studies showed that using 0.5 M HCl was a good eluent for copper recovery (89%).

Jaafarzadeh et al. [36] synthesized chitin from extracted shrimp shells in order to test its ability to adsorb Zn^2+^. Batch experiments were carried out in order to examine the influence of pH (3–7), the initial Zn^2+^ concentration (50–500 mg/L) and the adsorbent dose (0.5–10 g in 250 mL) on metal uptake. The adsorbed amount in mg/g was found to increase by increasing the pH and the initial concentration while the opposite results were noticed with the increment of the adsorbent dose. FTIR spectra of raw and Zn-loaded chitin biomass suggested that functional groups such as amine (−NH_2_) and hydroxyl (–OH) had the highest effect on zinc adsorption. Chitin extraction from the exoskeleton of the crab was synthesized and used as an adsorbent for Zn^2+^ [37]. The highest adsorption occurred at pH 7 (studied pH range 3–7), and at solid:liquid ratio of 1:500 g/mL (studied dosage range 1:500–20:500 g/mL). Equilibrium was achieved after 180 min. Zn^2+^ removal by chitin was also explored by Kocer et al. [38]. Batch experiments were carried out in terms of the effect of pH, temperature, contact time and initial concentration. The best adsorption was 8.21 mg/g at a 300 mg/L initial concentration, pH 4.5 and at 40 °C. A thermodynamic study demonstrated that the adsorption was spontaneous and endothermic with an increase in randomness at the solid-solution interface during adsorption. Chitin was produced from shrimp carapaces with the aim to remove Zn^2+^ [39]. Maximum adsorption appeared at pH 7 and equilibrium was achieved in 6 h. The increase of the temperature from 10 to 30 °C positively affected the Zn^2+^ uptake, while with a further increase up to 40 °C, a reduction of adsorption was observed. Batch experiments also revealed that the biosorption of Cu^2+^, Cd^2+^ and Zn^2+^ followed the order: Cu^2+^ > Cd^2+^ > Zn^2+^.

Chitin extracted from shrimp shells was also examined for As^5+^ adsorption [40]. The best adsorption was noticed at pH 4 and the removal was rapid in the first 30 min, during which 40%–50% of the arsenate was removed. Equilibrium was attained in 120 min. FTIR spectra before and after adsorption confirmed that the –CH_3_, –OH and –NH_2_ groups were involved in capturing the arsenate. Unmodified chitin nanofibers and thiol-modified chitin nanofibers were fabricated and tested to remove As^3+^ [41]. The modified chitin had a higher removal capacity, i.e., 138 mg/g at pH 7.0 instead of 58 mg/g at pH 5, which was given by untreated chitin nanofibers. One possible explanation is that the high concentration of thiol led to the formation of the As(III)-thiolate complex on the chitin’s nanofiber surface. Modified chitin was also synthesized and tested to adsorb As^3+^ [42]. More specifically, an acrylonitrile monomer was doped onto chitin (deacetylated form) using the pre-irradiation method (deacetylation degree (DDA) ~40%); then –CN groups were transformed into –C(NH_2_)=N–OH groups after modification with NH_2_OH. The BET surface area was estimated to be 1.278 m^2^/g. The maximum adsorption capacity was determined to be 19.724 mg/g.

Singh and Nagendran [43] used chitin to remove Cr^3+^. The adsorption was controlled by pH and the best adsorption was given at pH 3 and a maximum removal of 49.98% was presented in 20 min. The Langmuir isotherm model exhibited higher regression coefficients than Freundlich (0.915 vs. 0.831). Santosa et al. studied the adsorption of Cr^3+^ by chitin-humic acid hybrid (chitin-HA) from both synthetic and real samples of tannery wastewater treatment’s effluents [44]. Based on FTIR spectra before (raw material) and after adsorption (Cr-loaded material), it was found that –COO^−^ and *N*-acetyl originating from, respectively, humic acid and chitin participated in the adsorption of Cr^3+^. The application of chitin-HA in real wastewater treatment’s effluent showed that the maximum amount of Cr^3+^ using 1 g chitin-HA was 2.08 × 10^−4^ mol, equivalent to 10.82 mg.

The uptake of Cr^6+^ by Bargi scale and from chitin obtained from Bargi fish (*Heterotis Miloticus*) was examined [45]. The highest removal was found at pH 6–8 and chitin gave better adsorptive results than fish scale. The estimated thermodynamic parameters demonstrated the exothermicity and the feasibility of the uptake process. In another work, a biopolymer of chitin/bentonite was used for Cr^6+^ uptake by Saravanan et al. [46]. Optimum removal was noticed at pH 4 (pH studied in the range of 1–8). The adsorption was raised when the adsorbent amount was increased from 0.2 to 1 g, while a declined of the adsorbed Cr occurred for adsorbent dosages higher than 1 g. A sharp increase of the adsorption percentage was observed at around 30 min and reached the maximum at 45 min. The Freundlich isotherm model was a better fit than the Langmuir isotherm model. Karthik et al. used polypyrrole-functionalized chitin to adsorb Cr^6+^ [47]. The results showed that with the increase of the pH from 2 to 10, a reduction of the adsorption percentage from 92.34% to 15.07% was noticed. The authors explained that (i) at alkaline pH values, an antagonistic effect between –OH^−^ and Cr^6+^ ions and (ii) repulsion among chromate ions and the negative surface of the adsorbent occurred. Moreover, the removal percentage was increased by the increment of the adsorbent dosage from 0.05/50 to 0.25/50 mL. The presence of ions such as SO_4_^2−^, HCO_3_^−^ and NO_3_^−^ negatively affected the chromium removal suggesting competitive relation (antagonism).

Shao et al. modified chitin in THF (tetrahydrofuran) with l-cysteinein in the presence of sulfuric acid as a catalyst, in order to adsorb Cu^2+^, Cd^2+^, Pb^2+^, Zn^2+^, and Ni^2+^ [48]. The removal capacity for Cu^2+^, Cd^2+^, Pb^2+^, Zn^2+^ was 86.1, 214.6, 351.5, 107.0 mg/g and 57.9, 108.0, 132.4, 46.9 mg/g for cys-chitin and chitin, respectively, suggesting the success of the modification process. The cys-chitin appeared to have a higher affinity towards Zn^2+^, Cd^2+^, and Pb^2+^ following the order Zn^2+^ > Cd^2+^ > Pb^2+^. The maximum adsorption for all tested metals was noticed at neutral pH values (pH 6–7), except for Cu^2+^ which gave a maximum at 5.0. Desorption studies (six cycles of adsorption/desorption) indicated that loaded metal ions on cys-chitin can be desorbed completely by using 0.5 M HNO_3_. Moreover, the regeneration studies showed that the material deterioration was minimal. Table 1 summarizes all the above information, while Table 2 summarizes the respective adsorption experimental conditions.

### Thermodynamic Studies

In Table 3, the estimated thermodynamic parameters of Gibbs free energy (Δ*G*^0^) (Equation (1)), enthalpy change (Δ*H*^0^) and entropy change (Δ*S*^0^) for the adsorption of toxic metals by chitin-based adsorbents are listed. The Δ*G*^0^, Δ*H*^0^ and Δ*S*^0^ are linked with Equations (2) and (3). As can be seen, the sorption data ranged from three or four temperatures, used in the range of 288–323 K. Thermodynamic studies showed that the adsorption was spontaneous or non-spontaneous (Δ*G*^0^ < 0, Δ*G*^0^ > 0, absolute value 0.23–76.54 kJ/mol), endothermic or exothermic (Δ*H*^0^ < 0, Δ*H*^0^ > 0, absolute value 2.16–132.59 kJ/mol), with positive or negative entropy values (absolute value 0.01–0.185 kJ/mol·K).

(1) $$\Delta G^{0}=-RT\ln Κ$$

(2) $$\Delta G^{0}=\Delta H^{0}-T\Delta S^{0}$$

(3) $$\Delta S^{0}=-\frac{\Delta G^{0}}{T}-\left(-\frac{\Delta H^{0}}{T}\right)$$

**Figure 3:**
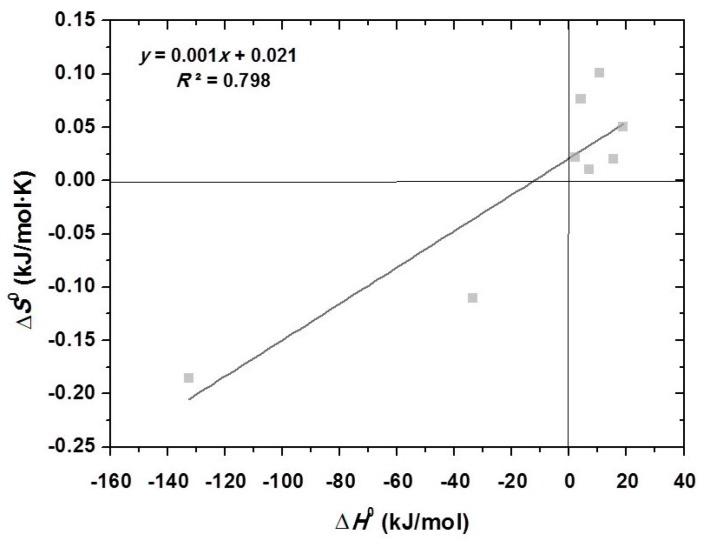
The enthalpy-entropy compensation plot for the works studied in the present review article (eight experimental points included).

Based on Equation (3), a plot of Δ*H*^0^ versus Δ*S*^0^ was drawn and a strong linear relationship was observed (*R*^2^ = 0.80) (Figure 3), which is known as enthalpy-entropy compensation [49,50,51,52,53,54,55,56].

This phenomenon sounds strange and it is hard to explain since the results came from various studies with different experimental conditions. One possible explanation is that both Δ*H*^0^ and Δ*S*^0^ were calculated from the same equation. To avoid misunderstandings, thermodynamic data should be viewed with caution.

## 4. Conclusions

This review focuses on the removal of toxic metals by chitin-based adsorbents. The initial pH, initial concentration, adsorbent dosage, contact time, etc., can also significantly affect the overall adsorption process. The Langmuir isotherm model and pseudo-second-order model were found to fit better to the adsorption data. More works suffer from the fact that there is a lack of discussion of the adsorption mechanism. For this purpose, in order to explore the mechanism of adsorption in depth, it is recommended to examine three-parametric isotherm models apart from two-parametric ones (Langmuir, Freundlich, etc.). Moreover, Weber and Morris and Elovich kinetic models are essential. Thermodynamic studies should also be viewed with caution since an enthalpy-entropy compensation was noticed. Future work should focus on a cost analysis of the application of chitins as adsorbents and their application in real wastewaters under real environmental conditions.

## Figures and Tables

**Table 1:** List of models for adsorption isotherms and kinetics for adsorption of metals on chitin-based adsorbents.

Chitin Adsorbent	Metal	Isotherm Model	Kinetic Model	*Q*_m_ (mg/g)	Reference
Chitin	Fe^3+^	L, F	-	1.3778	[29]
Chitin of pink shrimp	Pb^2+^	F	PS2	7.003	[30]
Chemically modified chitin with polypyrrole	Pb^2+^	F	PS2	8.64	[31]
Chemically modified chitin with polypyrrole	Cd^2+^	F	PS2	6.17	[31]
Chitin	Cd^2+^	L	-	90.1	[32]
Chitin/polyethylene glycol binary blend	Cd^2+^	F	PS2	156 ^a^	[33]
Chitin	Cd^2+^	L	-	32.4	[34]
Calcareous chitin	Cd^2+^	L	-	71.4	[34]
Chitin	Cu^2+^	L	PS2	58	[35]
Chitin from extracted shrimp shells	Zn^2+^	F	PS2	270.270	[36]
Chitin from extracted from exoskeleton of crab shells	Zn^2+^	F	PS2	181.18	[37]
Chitin	Zn^2+^	L	PS2	4.65	[38]
Chitin from shrimp carapaces	Zn^2+^	L	-	5.86	[39]
Chitin extracted from shrimp shells	As^5+^	F	PS1, PS2	11.574	[40]
Thiol-modified chitin nanofibers	As^3+^	L	-	149	[41]
Modified chitin	As^3+^	L	-	19.724	[42]
Chitin	Cr^3+^	L	-	7.738	[43]
Chitin-humic acid hybrid	Cr^3+^	F	FO	9.2	[44]
Bargi scale	Cr^6+^	L	-	25	[45]
Chitin from Bargi fish (*Heterotis Miloticus*)	Cr^6+^	L	-	37.04	[45]
Polypyrrole-functionalized chitin	Cr^6+^	F	PS2	28.92	[47]

^a^ *Q*_m_ obtained from kinetic adsorption studies; L: Langmuir; F: Freundlich; PS1: Pseudo-first-order kinetic model; PS2: Pseudo-second-order kinetic model; FO: First-order kinetic model.

**Table 2:** Adsorption experimental conditions of the studies summarized.

Chitin Adsorbent	Metal	Experimental Adsorption Conditions	Reference
Chitin	Fe^3+^	*C*_0_ = 2–14 mg/L; *m* = 10 mg; pH = 2–8; *V* = 50 mL; *T* = 20–50 °C	[29]
Chitin of pink shrimp	Pb^2+^	*C*_0_ = 1–20 mg/L; *m* = 150 mg; pH = 2–10; *V* = 50 mL; *T* = 30 °C	[30]
Chemically modified chitin with polypyrrole	Pb^2+^	*C*_0_ = 10 mg/L; *m* = 100 mg; pH = 2–6; *V* = 50 mL; co-existing ions: Cu(II), Zn(II), Co(II), Ni(II)	[31]
Chemically modified chitin with polypyrrole	Cd^2+^	*C*_0_ = 10 mg/L; *m* = 100 mg; pH = 2–6; *V* = 50 mL; co-existing ions: Cu(II), Zn(II), Co(II), Ni(II)	[31]
Chitin	Cd^2+^	*C*_0_ = 0.1 g/mL; *m* = 30 mg; pH = 2–10; *V* = 30 mL; *T* = 25–45 °C	[32]
Chitin/polyethylene glycol binary blend	Cd^2+^	*C*_0_ = 200 mg/L; *m* = 1–6 g; pH = 4–8; *T* = 30 °C	[33]
Chitin	Cd^2+^	*C*_0_ = 0–474 mg/L; *m* = 50 mg; pH = 3–7; *V* = 50 mL; *T* = 20 °C; [NaClO_4_] = 10^−1^; 10^−2^ and 10^−3^ M	[34]
Calcareous chitin	Cd^2+^	*C*_0_ = 0-474 mg/L; *m* = 50 mg; pH = 3–7; *V* = 50 mL; *T* = 20 °C; [NaClO_4_] = 10^−1^; 10^−2^ and 10^−3^ M	[34]
Chitin	Cu^2+^	*C*_0_ = 150–300 mg/L; *m* = 0.2 g; pH = 7; *V* = 100 mL; *T* = 25–45 °C	[35]
Chitin from extracted shrimp shells	Zn^2+^	*C*_0_ = 50–500 mg/L; *m* = 0.5–10 g; pH = 3–7; *V* = 250 mL; *T* = 25 °C	[36]
Chitin from extracted (exoskeleton) crab shells	Zn^2+^	*C*_0_ = 50–500 mg/L; *m* = 0.5–10 g; pH = 3–7; *V* = 250 mL; *T* = 25 °C	[37]
Chitin	Zn^2+^	*C*_0_ = 10–300 mg/L; *m* = 0.1 g; pH = 2–5; *V* = 100 mL; *T* = 20–40 °C	[38]
Chitin from shrimp carapaces	Zn^2+^	*C*_0_ = 20–400 mg/L; *m* = 0.1–0.6 g; pH = 2–7; *V* = 300 mL; *T* = 10–40 °C	[39]
Chitin extracted from shrimp shells	As^5+^	*C*_0,As_ = 0.5–10 mg/L; *C*_0,Zn_ = 50–500 mg/L; *m* = 0.1–10 g; pH = 3–7; *V* = 500 mL; *T* = 10–40 °C	[40]
Thiol-modified chitin nanofibers	As^3+^	*C*_0_ = 10–100 mg/L; *m* = 0.5 wt %; pH = 4–11; *T* = 25 °C	[41]
Modified chitin	As^3+^	*C*_0_ = 65–650 mg/L; *m* = 3 g; pH = 6.5; *T* = 30 °C	[42]
Chitin	Cr^3+^	*C*_0_ = 50 mg/L; *m* = 0.1 g; pH = 2–5; *V* = 50 mL	[43]
Chitin-humic acid hybrid	Cr^3+^	*C*_0_ = 50 mg/L; *m* = 10 mg; pH = 3–7; *V* = 10 mL; *T* = 25 °C	[44]
Bargi scale	Cr^6+^	*C*_0_ = 0–100 mg/L; *m* = 100 mg; pH = 3–8; *V* = 50 mL; *T* = 30 °C	[45]
Chitin from Bargi fish (*Heterotis Miloticus*)	Cr^6+^	*C*_0_ = 0–100 mg/L; *m* = 100 mg; pH = 3–8; *V* = 50 mL; *T* = 30 °C	[45]
Polypyrrole-functionalized chitin	Cr^6+^	*C*_0_ = 50–100 mg/L; *m* = 0.1 g; pH = 4.8; *V* = 50 mL; *T* = 30–50 °C	[47]

**Table 3:** Thermodynamic parameters estimated for the adsorption of different metals onto chitin-based adsorbents.

Chitin Adsorbent	Metal	*T* (K)	Δ*G*^0^ (kJ/mol)	Δ*Η*^0^ (kJ/mol)	Δ*S*^0^ (kJ/mol K)	Reference
Chitin	Fe^3+^	293	−4.32	2.16	0.022	[29]
		303	−4.52			
		313	−4.72			
		323	−4.97			
Chemically modified chitin with polypyrrole	Pb^2+^	303	−3.03	18.92	0.05	[31]
		313	−2.84			
		323	−1.98			
Chemically modified chitin with polypyrrole	Cd^2+^	303	−4.61	7.19	0.01	[31]
		313	−4.62			
		323	−4.44			
Chitin	Cd^2+^	288	−18.5	10.6	0.101	[32]
		298	−19.5			
		308	−20.5			
		318	−21.5			
Chitin	Cu^2+^	298	−0.87	−33.65	−0.11	[35]
		308	0.23			
		318	1.33			
Chitin	Zn^2+^	293	−18.21	4.13	0.076	[38]
		303	−18.92			
		313	−19.73			
Chitin from Bargi fish (*Heterotis Miloticus*)	Cr^6+^	–	−76.54	−132.59	−0.185	[45]
Polypyrrole-functionalized chitin	Cr^6+^	303	−8.30	15.51	0.02	[47]
		313	−8.17			
		323	−7.82			
